# Correction: The application of ICG-based photodynamic therapy combined with nanotechnology in tumor treatment

**DOI:** 10.1039/d6ra90036d

**Published:** 2026-04-08

**Authors:** Hexin Fu, Lu Zhao, Dong Tang

**Affiliations:** a Northern Jiangsu People's Hospital Affiliated to Yangzhou University Yangzhou 225000 China 431245408@qq.com; b The Yangzhou Clinical Medical College of Xuzhou Medical University Yangzhou 225001 China; c Department of General Surgery, Institute of General Surgery, Northern Jiangsu People's Hospital Affiliated to Yangzhou University, The Yangzhou Clinical Medical College of Xuzhou Medical University, The Yangzhou School of Clinical Medicine of Dalian Medical University, The Yangzhou School of Clinical Medicine of Nanjing Medical University, Northern Jiangsu People's Hospital, Clinical Teaching Hospital of Medical School, Nanjing University Yangzhou 225000 China 83392785@qq.com +86-18952783556

## Abstract

Correction for ‘The application of ICG-based photodynamic therapy combined with nanotechnology in tumor treatment’ by Hexin Fu *et al.*, *RSC Adv.*, 2026, **16**, 7204–7220, https://doi.org/10.1039/D5RA08557H.

The authors regret that the funding information was incorrectly shown in the Acknowledgements section of the original manuscript. The corrected funding acknowledgement is as shown below.

The authors disclose receipt of the following financial support for the research, authorship, and/or publication of this article: This work was supported by the Social Development Project of the Key R&D Plan of the Jiangsu Provincial Department of Science and Technology [no. BE2022773]; the Graduate Research-Innovation Project in Jiangsu Province [no. SJCX21_1644]; the Academic Science and Technology Innovation Fund for College Students [no. 202011117056Y]; the Social Development-Health Care Project of Yangzhou, Jiangsu Province [no. YZ2021075]; the High-Level Talent “Six One Projects” Top Talent Scientific Research Project of Jiangsu Province [no. LGY2019034]; and the Graduate Research-Innovation Project in Jiangsu Province [no. SJCX22_1816]. The funding bodies had no role in the design of the study; in the collection, analysis, and interpretation of the data; or in the writing of the manuscript.

The Royal Society of Chemistry apologises for these errors and any consequent inconvenience to authors and readers.

